# Open source intelligence and AI: a systematic review of the GELSI literature﻿

**DOI:** 10.1007/s00146-023-01628-x

**Published:** 2023-01-28

**Authors:** Riccardo Ghioni, Mariarosaria Taddeo, Luciano Floridi

**Affiliations:** 1 Department of Legal Studies, University of Bologna, Via Zamboni, 27, 40126 Bologna, IT Italy; 2 Oxford Internet Institute, University of Oxford, 1St Giles’, Oxford, OX1 3JS UK; 3 The Alan Turing Institute, British Library, 96 Euston Rd, London, NW1 2DB UK

**Keywords:** Artificial intelligence, Digital ethics, GELSI, Machine learning, Open source intelligence, OSINT

## Abstract

Today, open source intelligence (OSINT), i.e., information derived from publicly available sources, makes up between 80 and 90 percent of all intelligence activities carried out by Law Enforcement Agencies (LEAs) and intelligence services in the West. Developments in data mining, machine learning, visual forensics and, most importantly, the growing computing power available for commercial use, have enabled OSINT practitioners to speed up, and sometimes even automate, intelligence collection and analysis, obtaining more accurate results more quickly. As the infosphere expands to accommodate ever-increasing online presence, so does the pool of actionable OSINT. These developments raise important concerns in terms of governance, ethical, legal, and social implications (GELSI). New and crucial oversight concerns emerge alongside standard privacy concerns, as some of the more advanced data analysis tools require little to no supervision. This article offers a systematic review of the relevant literature. It analyzes 571 publications to assess the current state of the literature on the use of AI-powered OSINT (and the development of OSINT software) as it relates to the GELSI framework, highlighting potential gaps and suggesting new research directions.

## Introduction

Literature about intelligence studies claims that open source intelligence (OSINT), i.e., intelligence derived from publicly available sources, makes up between 70 and 90 percent of all contemporary intelligence material (Hulnick 2002, 566; Unver 2018, 5). This estimate is not surprising as open-source information increases and more efficient techniques from computer science, data science, and statistics are developed, streamlining collection and analysis. As capabilities grow with the development of artificial intelligent (AI) systems, performance becomes inextricably linked to the quality of the technical tools employed by OSINT analysts. As a result, important issues related to the governance of these developments arise in both academic and applied domains. Indeed, it has become crucial to devise appropriate legal, ethical and regulatory frameworks to tackle the challenges posed by the increasing complexity of AI systems as they interact with every stage of the OSINT cycle—direction, collection, processing, analysis, dissemination and integration, and feedback (Defense Technical Information Center (DTIC)—Department of Defense 2013).^1^ Some earlier work, taking note of this trend, has provided an overview of the use of AI algorithms for OSINT analysis in the applied literature (Evangelista et al. 2021), while other authors have focused on the impact of the General Data Protection Regulation (GDPR) on the collection and analysis of OSINT (Shere 2020b). So far, however, a thorough review of the Governance, Ethical, Legal and Social Implications (GELSI)^2^ framework applied to OSINT is still lacking. This article sets out to fill this gap by providing a systematic review of the OSINT-GELSI literature as defined in Grant and Booth (2009, 102), namely a systematic search and analysis of the relevant literature. This is achieved by collecting a bibliographic dataset of OSINT articles which is then vetted to identify articles dealing with the GELSI framework. Current research is then summarized according to its major underlying themes, and some novel research directions are suggested.

The article is structured as follows. Section two provides more detailed definitions of OSINT and presents a brief historical overview of its scope and applications over the years. We argue that, because of the digital revolution, OSINT capabilities have been greatly increased in terms of data availability and computational power. We also provide a working definition of the GELSI framework and how it relates to current research on AI auditing and regulation. Section three details the methodology used to retrieve the bibliographic dataset and presents the results of a bibliometric analysis conducted on the different strands of OSINT literature. It shows that, despite a vast increase in publications, papers dealing with the GELSI framework are still a small subset of the wider scholarship, with technical papers dealing with OSINT collection and analysis being the largest group. However, it also indicates that, once accounting for the low publication numbers, GELSI papers have become increasingly more influential over the years, both in terms of citation counts and ranking in search engines. Sections four to six provide a systematic review of the relevant literature in terms of GELSI, highlighting the main themes underpinning most of the reviewed material. Section seven then suggests future research directions concerning the role of AI-augmented OSINT systems. Finally, section eight summarizes the main findings and concludes the article.

## Open source intelligence, AI, and the GELSI framework

A great deal of literature on OSINT has been devoted to finding a suitable definition for it. This is not easy because the concept of intelligence analysis is still contested in the relevant literature (Ish et al. 2021), with different authors and institutions providing different definitions, and because any definition of OSINT needs to accommodate advances in computer and data science and AI, which are constantly expanding the intelligence collection and analysis capabilities. One of the earliest definitions is found in the Intelligence Community Directive 301, a document aimed at increasing awareness of open-source information among intelligence agencies. Directive 301 borrowed its definition of OSINT from Public Law 109-163 (or the National Defense Authorization Act of 2006), stating that:

Open-source intelligence (OSINT) is intelligence that is produced from publicly available information and is collected, exploited, and disseminated in a timely manner to an appropriate audience for the purpose of addressing a specific intelligence requirement

(Public Law 109-163 2006, Division A, Title IX, Subtitle D, Sec. 931)

This definition is quite broad and does not detail the wide range of OSINT applications. Indeed, for most of its early history, OSINT has been limited to the physical retrieval and analysis of foreign media by offices such as the United States (US) Foreign Broadcast Information Service (FBIS), which was tasked with listening to, translating, and analyzing Axis broadcasts, to gain strategic information about the enemy’s intentions (Mercado 2001). This was the so-called first generation of OSINT, whose main tasks were document retrieval and translation and required little analytic work aside from some content analysis on the collected material (Williams and Blum 2018, 40)*.*

The landscape changed dramatically at the turn of the century. The creation of the Open Source Center (OSC) in 2005, which replaced the FBIS in the US, marks the beginning of the second generation of OSINT, whose crucial innovations were made possible mainly by the digital revolution. As observed by Unver (2018), the shift from “classical” to “digital” OSINT unlocked powerful and previously unthinkable tools, which can be roughly divided into four major groups, namely linguistic and text-based, geospatial, network-based, and visual forensics.

Linguistic tools relate to the retrieval and analysis of textual data and constitute a clear bridge between the first and the second generations of OSINT. If the former had analysts sifting through documents to detect valuable information and produce executive summaries, the latter saw computer algorithms scanning digitized documents to extract keywords and identify their context. Natural Language Processing (NLP) is a discipline at the crossroads of linguistics and AI, dealing with the analysis of textual data in different domains. Many algorithms designed to solve a wide variety of problems in machine learning—such as topic discovery, entity recognition, and automatic text summarization (Unver 2018, 8)—have been applied, together with information retrieval algorithms, to analyze open-source information gathered from online newspapers and social media. This has enabled researchers to sort rapidly through large pools of data and identify semantic patterns, translate and summarize long documents and detect behavioral changes through sentiment analysis (Chen 2011; Neri et al. 2012; Asghar et al. 2015).

Geospatial tools refer to any method through which open-source information directly or indirectly situates an actor or a group of actors in space. The emergence of commercial satellite imagery and other remote sensing tools has made geospatial OSINT very popular among analysts, who can now overlay locations mined from the web with satellite images, visualizing movements over time, and connections between locations (Unver 2018, 11). Applications of geospatial OSINT include *geolocation, geo-inference*, i.e., retrieving users’ locations without explicit geotagging information, and *georeferencing*, namely uniquely identifying geographical objects (Williams and Blum 2018, 33).

Network-based tools involve using measures borrowed from network science*,* a discipline studying pairwise relationships between entities. Social network data make for a great source of OSINT since relationships can be easily harvested and mapped, identifying the strength of relationships between actors (Unver 2018, 12). Centrality measures can then be computed for the entities in a network, allowing analysts to quantify the relative importance of each unit in regulating the information flow through the group. These tools have found important applications in studying terrorist networks (Wiil 2011), and they are increasingly exploiting the enormous quantity of online social network data to obtain more accurate estimates.

Finally, visual forensics tools are techniques for extracting valuable information from image and video files (Unver 2018, 13–14). For instance, metadata stored under the Exchangeable Image File Format (EXIF) in digital cameras and smartphones can yield crucial intelligence, such as the date, time, and location where the file was created. Moreover, tools for detecting doctored images and conducting photogrammetric analysis (the acquisition of measurements from photographs and videos) are also available to the OSINT analyst.

The increasing reliance on AI to automate most of the collection and analysis process foresees the emergence of a third-generation OSINT, more dependent on computer algorithms and automated reasoning than on the analyst’s supervision (Williams and Blum 2018, 39–40).

The above historical excursus shows how difficult it has become to provide a unified definition of OSINT. Indeed, unlike other intelligence-gathering disciplines, OSINT is aided by developments in the digital world, and its domain expands with them. As new data sources become widely available, new, previously hidden patterns can be learned from them, further blurring the lines between different intelligence practices [see, for instance, the discussion on a possible expansion of OSINT to the augmented reality domain in Williams and Blum (2018)].

Yet, despite these difficulties, the above presentation suffices to illustrate the far-reaching possibilities of OSINT and introduce our work. In the following pages, we shall review the literature dealing with the GELSI of second and third-generation OSINT. Although a formal definition of the GELSI framework is yet to be formulated, we take a broad approach (as the keyword specification in section three shows) and regard as GELSI-related literature any article tackling the meaningful changes or potential harms brought about by the use of AI-powered OSINT, together with the proposed solutions to such issues. The following section explains how we proceeded.

## Methodology

To create our bibliographic dataset, we used *Publish or Perish* (PoP, Harzing 1997), a software that allows researchers to query multiple academic databases and export the resulting reference list to conduct analysis. We queried the two main scholarly databases, *Google Scholar* and *Scopus.* To include as much material as possible, we only required the phrase “Open Source Intelligence” or its acronym “OSINT” in the paper’s title. In a separate search, we specified the same criterion for the phrase “Open Source Information” and its acronym “OSINF”. We ran the same two queries on both databases, for a total of four queries. Table 1 summarizes the results of this search, together with the number of results provided by *PoP.* After exporting the datasets, these were joined and scanned to remove both within and between-platform duplicates and works deemed irrelevant to the current analysis. These included documents, such as Master’s theses, conference talks, executive summaries and other papers that contained the search terms but in a different, unrelated context.

**Table 1:** Summary of results from the PoP queries

Database	Query	Entries returned	Entries included
Google Scholar	Intitle: Open Source Intelligence OR OSINT	625	440 (54%)
	Intitle: Open Source Information OR OSINF	185	
Scopus	Intitle: Open Source Intelligence OR OSINT	167	131 (60%)
	Intitle: Open Source Information OR OSINF	50	
Total	4	1027	571 (55%)

As can be seen in the right columns, the number of entries that were eventually removed is high in both databases. Once eliminated, this left 571 papers or around 55% of the original dataset. As a final step in the data cleaning and collection process, we crawled the web for the papers’ abstracts. *PoP* provides an abstract entry in its bibliographic files, but abstracts are available only for entries taken from *Google Scholar* and are only previews downloaded from the search results page. Therefore, a parsing script was designed to retrieve the abstracts’ text based on each article’s Digital Object Identifier (DOI). Since the HTML structures of the DOIs’ landing pages is quite varied, the script only achieves a *retrieval precision* of 43.87%. Thus, when no abstract could be crawled, or the DOI was missing, the *PoP* abstract was kept instead.

After the initial data cleaning steps, we performed a keyword search routine to split the dataset into different literature strands. First, we iterated through each entry’s title, abstract, and journal name to identify those belonging to the Practitioner Literature*,* which we define as works dealing with the practical aspects of OSINT gathering, analysis and interpretation, with a specific focus on Digital OSINT. These documents cover a wide variety of topics, such as the development of efficient data mining techniques for OSINT gathering, the creation of OSINT platforms for social media intelligence, the optimisation of NLP algorithms for entity ranking and identification or the use of deep learning models for cyber threat classification from OSINT data. However, they have in common the applied nature of their research, focusing on algorithmic solutions for problems arising at each stage of the OSINT cycle.

Once the practitioner literature was removed from our dataset, we were left with what we define as the intelligence literature, namely those documents dealing with OSINT as a discipline. Once again, this is a broad categorization, including, among other topics, historical accounts of the emergence and evolution of the discipline, case studies on OSINT applications and theoretical examinations of the advantages and disadvantages of employing OSINT over standard intelligence. From this area of the OSINT literature, we sought to extract any articles related to the GELSI framework. To do this, we performed a second keyword search on the papers’ titles, abstracts, and journal names. The list of keywords used to identify each strand, together with their locations, is provided in Table 2. Some of the keywords used are very specific (targeting specific papers identified before the keyword search). However, most are general and can be applied outside the OSINT corpus. This approach is not without issues. Indeed, some papers may lack any of the specified keywords in any of the fields, thus falling into the Intelligence category while belonging to one of the other two. Moreover, some entries may be of difficult classification, with their content not entirely fitting any of the above categories. However, a direct and careful inspection of the resulting dataset entries revealed that only a small fraction of papers was misclassified. This error was corrected. Most of the issues concerned only some minor overlap between the Practitioner and Intelligence strands, which are not the focus of the present review.

**Table 2:** List of keywords used to classify the bibliographic dataset

Classification	Title keywords	Abstract keywords	Journal/book keywords	Number of papers
Practitioner literature	Analytics - adaptive resonance theory - algorithm - artificial intelligence - automated - automating - automation - big data - cloud computing - computing - clustering - counterterrorism - covid-19 - cyber - cyberattack - cyber threat intelligence - dark web - data mining - dataset - deep learning - detection - entity ranking - extraction - geofencing - geographic information system - gis - image recognition - information technology - landsat - lidar - latent dirichlet allocation - machine learning - maltego - mapping - mathematical - method - metric - mining - model - monitoring system - mpeg - named entity recognition - natural language - natural language processing - network analysis - network science - network threat - nlp - neural network - nuclear - password - platform - practitioner - proliferation - quantitative - reaper - scada - seedsminer - semantic - sentiment analysis - social engineering - social network intelligence - statistical - statistics - systematic collection - technique - telegram - textual data - tool - toolkit - tor - twitter - whatsapp	Adaptive resonance theory - automated - automating - automation - crawling - cyber - cybersecurity - data mining - dataset - deep learning - latent dirichlet allocation - maltego - mining - named entity recognition - natural language processing - nlp - neural network - scraping - statistical - unstructured text	Automating - computer science - cyber - electrical - electronic - engineering - information system - management - marketing - mathematical - mathematics - physics - software - statistic - threat	255
GELSI literature	Accountability - audit - auditing - bias - caper - concern - court - criminal proceeding - discrimination - education - environment - ethical implication - ethical - ethics - general data protection regulation - gdpr - governance - human right - identity - information security behavior - internet age - judicial - law - legal - legal implication - legislation - legislative - narrative - oversight - privacy - reliability - regulation - regulatory - right - scrutiny - social implication - society - surveillance - transparency - veracity - victim - violence	Accountability - audit - auditing - caper - ethical implication - general data protection regulation - gdpr - human right - judicial - legal implication - legislation - legislative - oversight - privacy - regulation - regulatory - social implication - transparency	Ethic - human right - law - legal - policy - regulation - regulatory - social	69
Intelligence literature	Remaining papers not included in the classifications above			247
Total	114	38	23	571

**Fig. 1:**
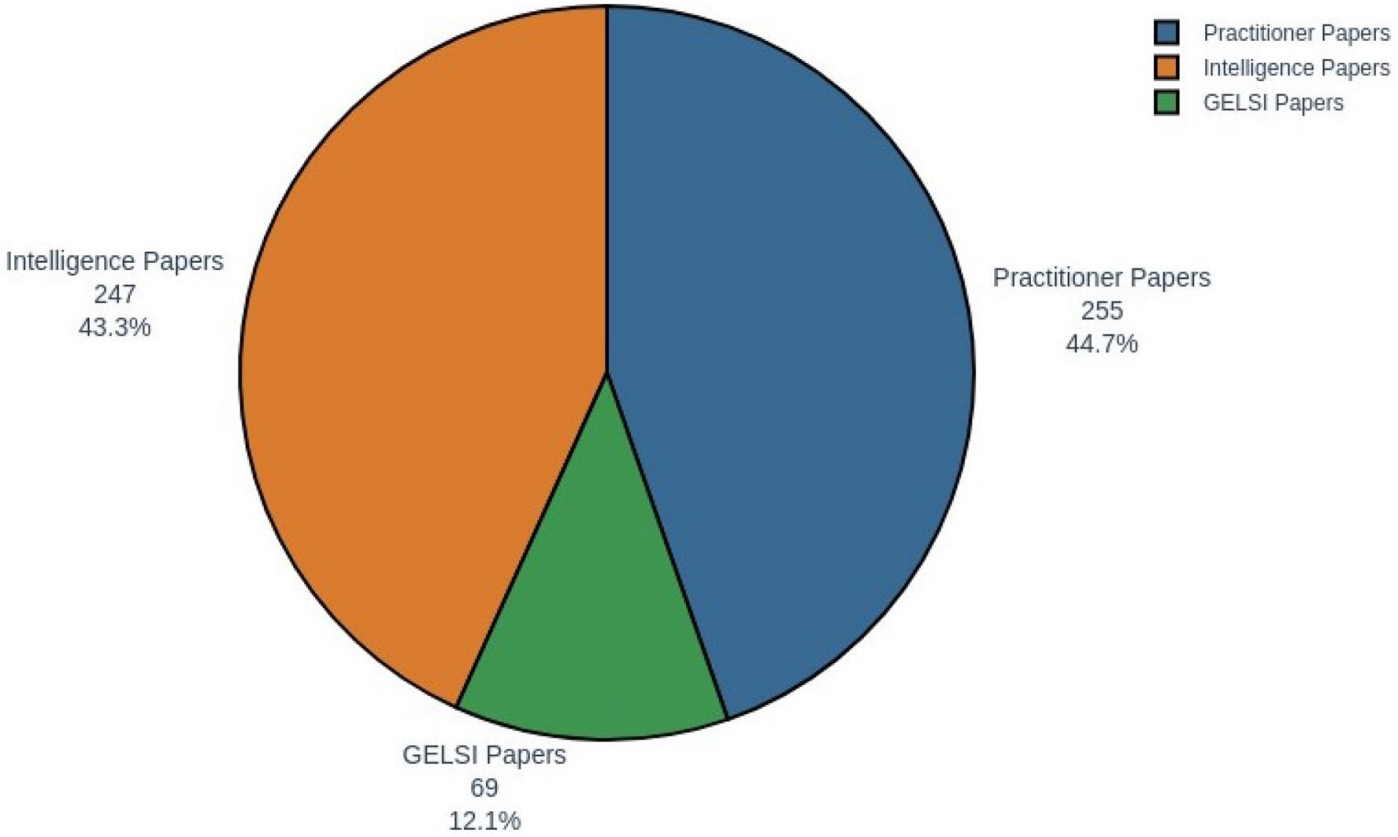
Distribution of literature areas

Once the keywords were specified and the literature strands returned, we plotted their percentage distribution in Fig. 1.

**Fig. 2:**
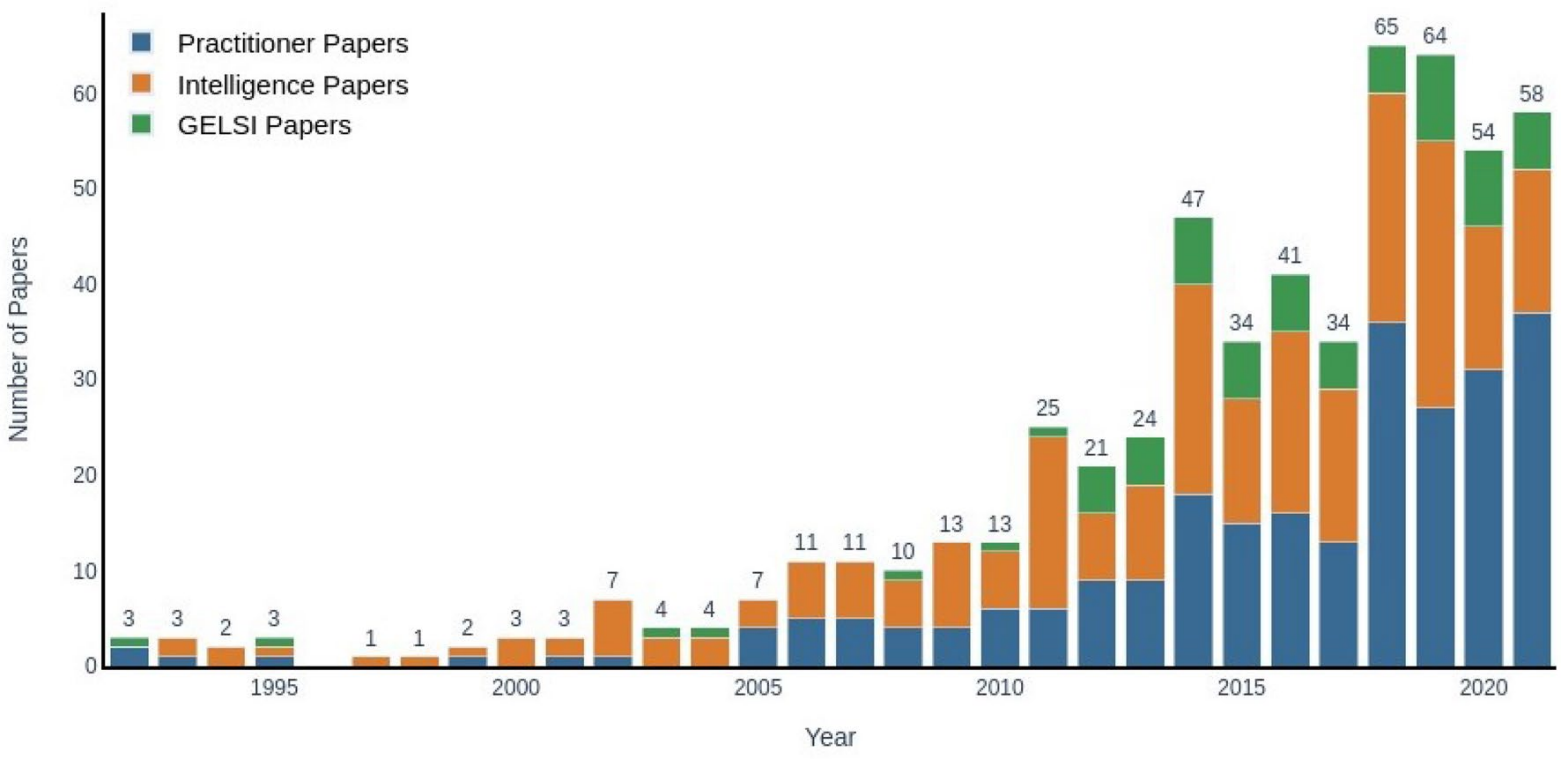
OSINT literature over the years (1992–2021)

GELSI-themed papers only account for about 12% of the entire corpus, while the remaining papers are evenly split between the practitioner and intelligence areas. In Fig. 2, we visualize the evolution of the OSINT literature over the last thirty years.

**Fig. 3:**
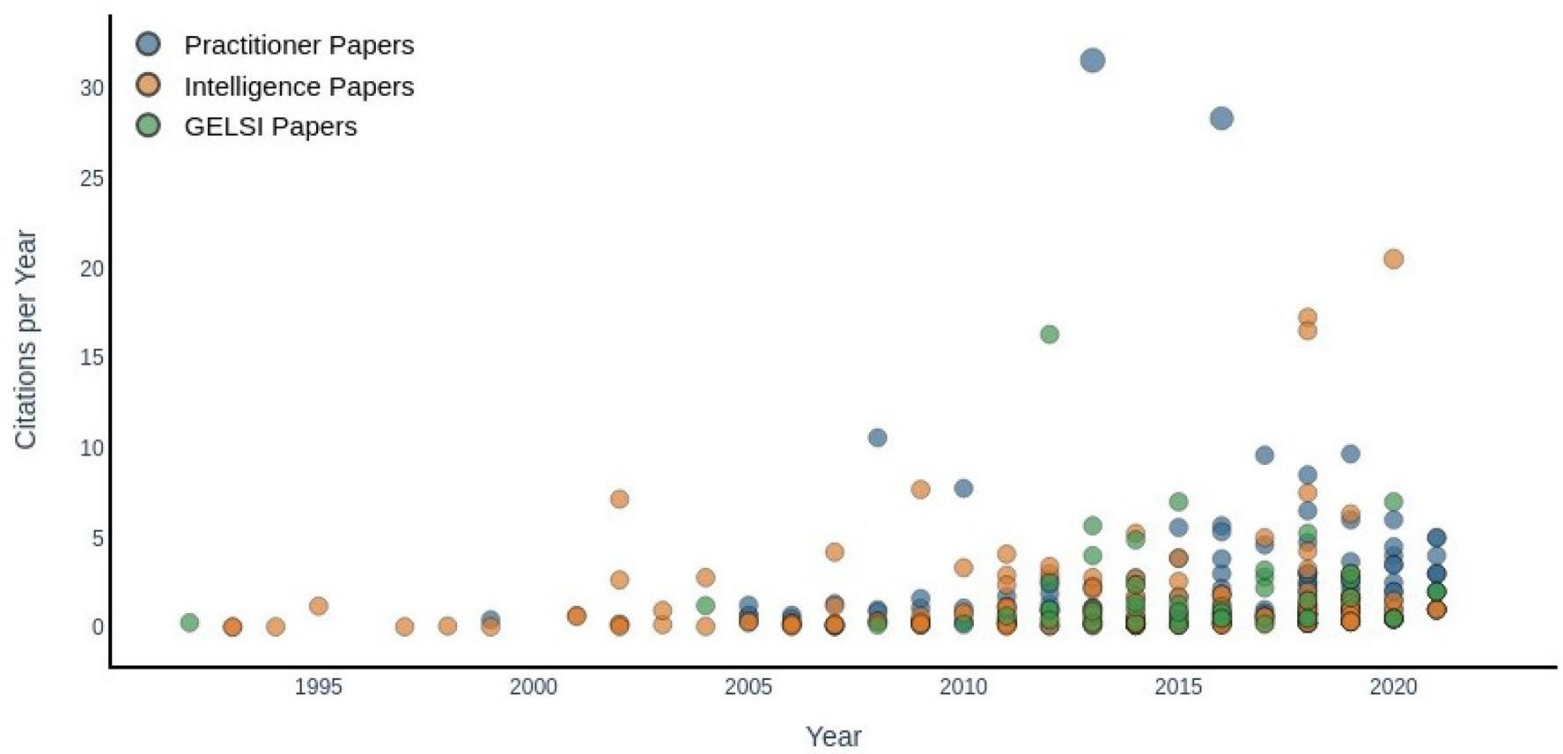
Scatter plot of paper citations

The number of published articles has increased dramatically since the early 1990s and especially in the last ten years. While the practitioner literature has witnessed the highest increase, GELSI scholarship has also grown significantly in recent years. This testifies to the perceived importance of developing up-to-date practical tools to deal with the ever-increasing pool of OSINT data and the need for viable ethical and legal frameworks to deal with such tools. Looking at the above graphs, it seems that this need has only been partially addressed. Indeed, GELSI scholarship appears to be only a minor subfield of the wider OSINT literature in terms of sheer publication numbers. However, one might also be interested in checking how influential each publication is to the others. Figure 3 plots the number of citations per year received by each paper, obtained by dividing the number of citations by the number of years elapsed since publication. Aside from a few very influential outliers in the practitioner and intelligence literature, most papers cluster around the same citation performance each year, irrespective of the research area. A slight upward trend can be detected in the last couple of years, which is compatible with the overall increase in the number of publications.

**Fig. 4:**
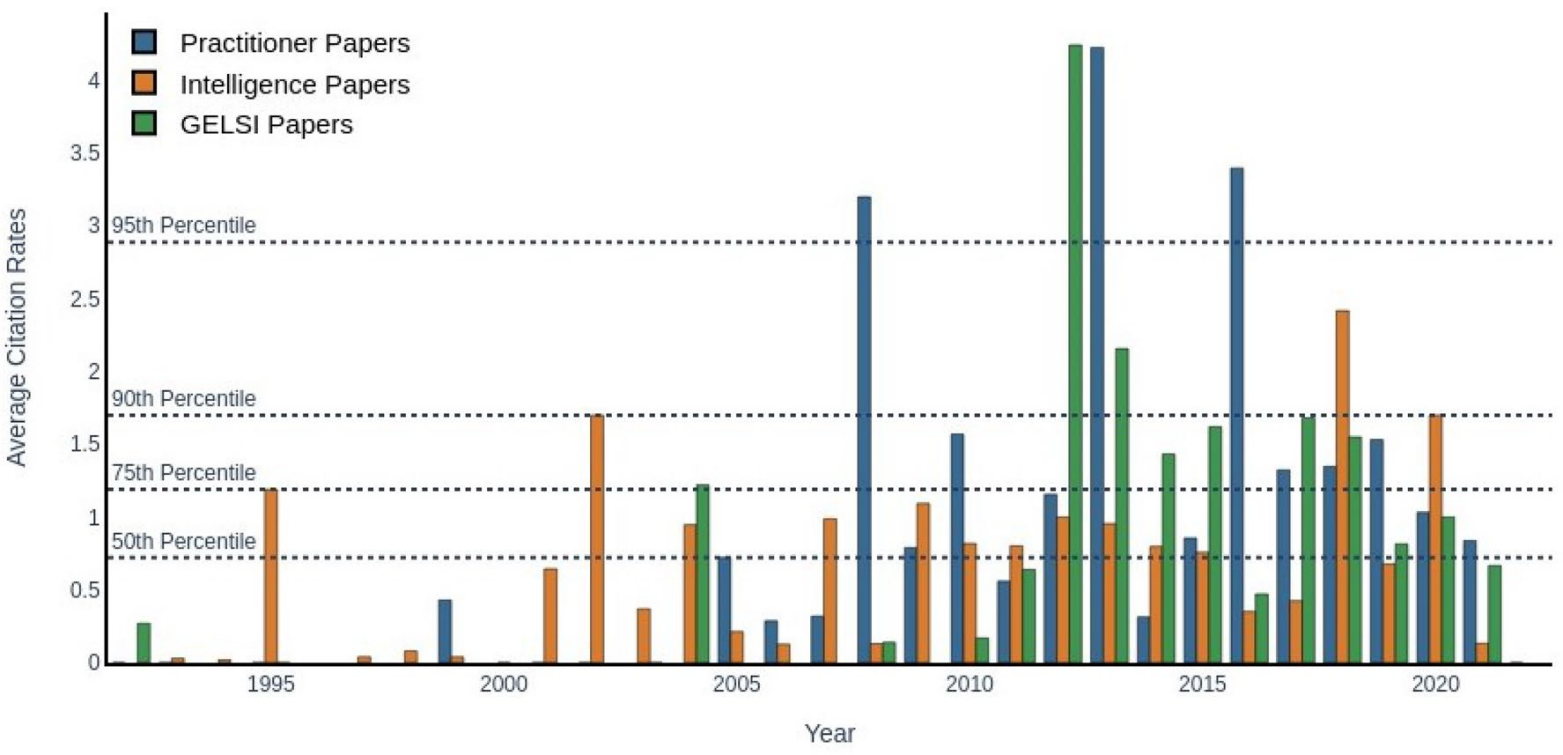
Average yearly citation rates by research area

Instead, if we consider each literature area as a separate corpus, i.e., we normalize citation rates by the number of publications in the same field each year, we obtain the average yearly citation rates, which we formally define as:

1 $$Z(t,f): = \frac{1}{N(t,f)}\sum\limits_{i = 1}^{N(t,f)} {c_{i} (t,f),}$$

where *T* = {1992, …, 2022}, $N\left(t,f\right)\in {\mathbb{N}}$ is the number of papers from field $f$ published in year $t$ and ${c}_{i}(t,f)$ is the number of citations paper $i\in \{1,\dots ,N(t,f)\}$ from field $f$ received in year $t$. Therefore, average yearly citation rates are functions of both year *t* and field *f*. These rates are plotted in Fig. 4.

This way, instead of the relative importance of specific papers, we obtain a rough estimate of the relative importance of each field and its evolution through the years. Although there is no clear trend detectable in the graph, we can see how GELSI papers suddenly stand out and appear to be highly influential, sometimes even more than papers in the remaining fields, despite being only 12% of the OSINT corpus.

**Fig. 5:**
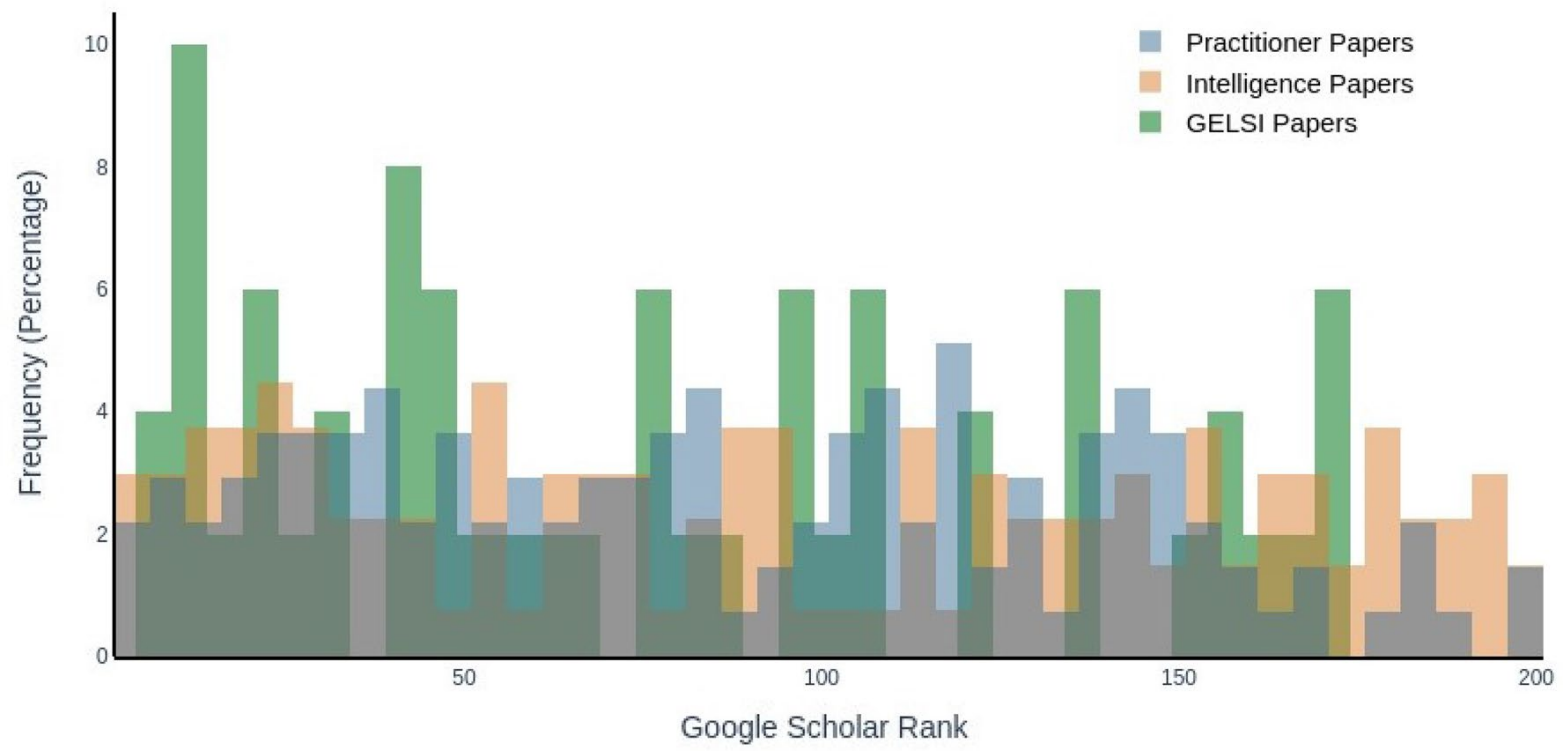
Frequency distribution of Google Scholar ranks

Finally, another measure that is useful in quantifying each field’s relative influence is the *Google Scholar* rank, namely the position of each paper in the Google Scholar queries. Figure 5 shows the frequency distribution of Google Scholar ranks for each subfield. Note that these ranks are automatically recorded by *PoP* after each of the queries in Table 1, and therefore they are not affected by any of the later keyword searches.

Indeed, while technical and intelligence articles have almost overlapping distributions of ranks, GELSI papers concentrate most of their values at the top, declining soon after. As it turns out, 72% of GELSI papers are found within the first 200 results, while the proportion drops to around 54% for the remaining literature fields. Computing the odds ratio, we discover that GELSI papers are twice more likely to be assigned a rank between 1 and 200 than papers belonging to any other field (*p* < 0.01).

It has been shown that citation counts play a major role in *Google Scholar*’s ranking algorithm (Beel and Gipp 2009). However, as the above plot and computations show, this is not the only metric considered when computing each paper’s rank. Indeed, other variables, such as the paper’s author and journal, also affect the ranking. The fact that a small subset of the OSINT corpus dealing with its ethical, legal, and social implications is more likely to be ranked higher than the remaining larger literature by one of the most prominent academic search engines testifies to the relevance of this area despite the low number of publications. Thus, it is paramount to investigate the OSINT-GELSI literature thoroughly and provide guidance on how researchers can further develop it.

Throughout the review, we also reference papers that do not belong to the bibliographic dataset described here either because they did not match any of the queries’ requirements or because they were found to help provide the necessary context to the topics discussed. Indeed, issues raised in other, maybe even loosely related fields could easily be applied to the OSINT landscape with much to gain in terms of a normative framework for modern OSINT applications. The following sections present the result of the analysis.

## Review of the OSINT-GELSI literature

**Fig. 6:**
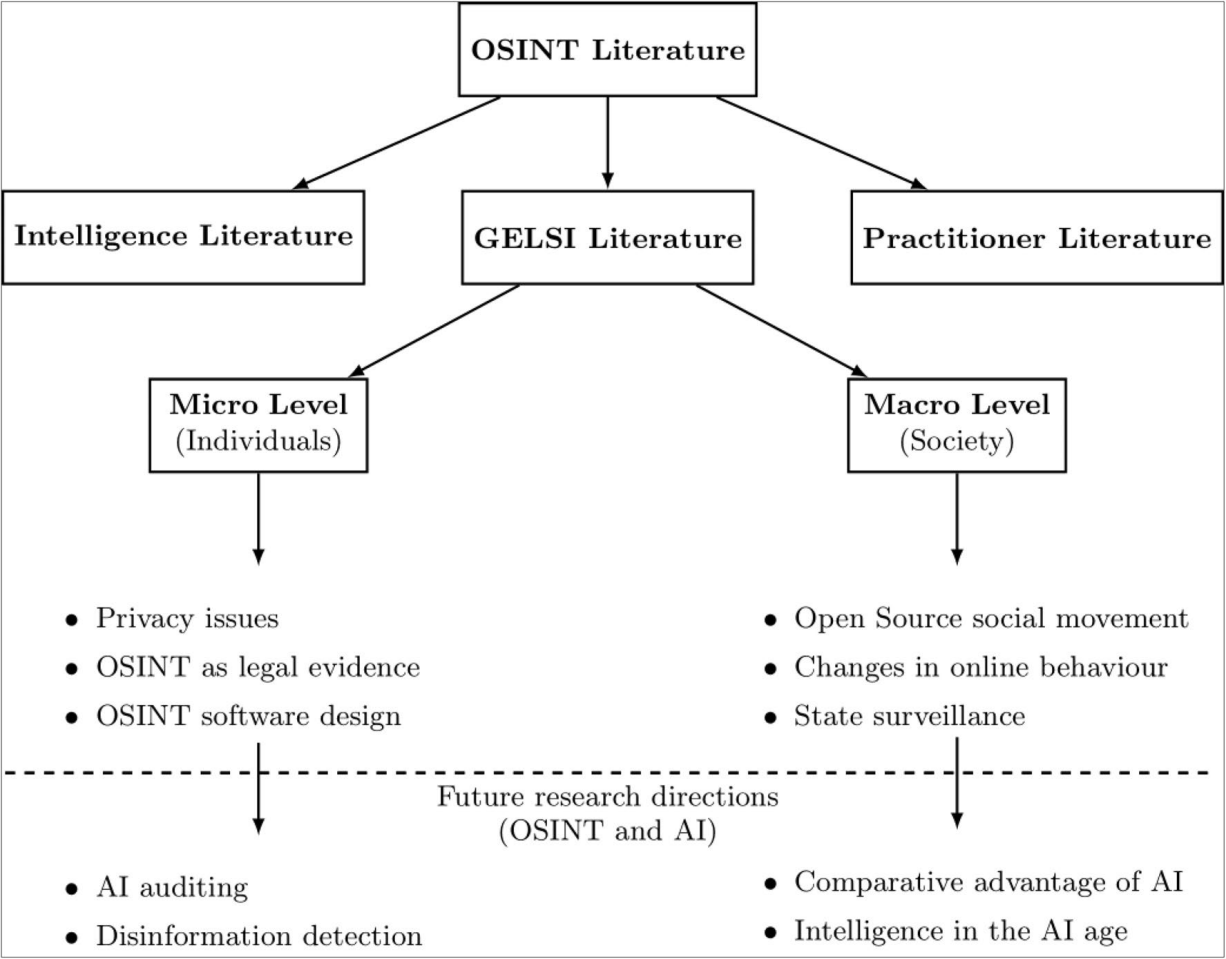
A proposed OSINT-GELSI taxonomy

Following the analysis of the relevant literature, Fig. 6 provides a proposed taxonomy of the GELSI scholarship in the context of the more comprehensive OSINT corpus, together with some of the most prominent themes for each area. As it turns out, we can distinguish two main levels of analysis.

The *micro-level* deals with the impact of OSINT on individuals and organizations, focusing on the legal and ethical challenges posed by the OSINT cycle, especially surrounding privacy issues emerging in the collection phase. We analyze papers addressing these aspects in Sect. 5.

The *macro-level* is concerned with the impact of OSINT on society at large. These papers focus on the social, governance and even behavioral implications of open-source information, addressing issues, such as the emergence of citizen activism and the changes in users’ online habits triggered by the mainstreaming of OSINT data and techniques. We examine these themes and highlight the main arguments put forward in these papers in Sect. 6.

Finally, in Sect. 7, we consider how the OSINT-GELSI literature will likely evolve, given the ever-increasing reliance on AI algorithms by OSINT analysts. At the micro-level, these emerging trends include the auditing of AI algorithms at the processing and dissemination stages of the OSINT cycle, while at the macro-level, they revolve around issues of asymmetric technological advantage and institutional accountability. Moreover, following Glassman and Kang (2012), we also look at OSINT as a problem-solving strategy, changing how users approach information, and focus on how AI is likely to influence this process.

## The GELSI of OSINT at the micro-level

The first, most prominent theme in the GELSI literature concerns the *micro implications* of second and third-generation OSINT. This scholarship mainly examines the legal and ethical aspects of open-source data gathering and analysis. Koops (2013) argues that the mere fact that some information is public does not mean privacy concerns should be discarded entirely. Moreover, modern OSINT techniques can aggregate several chunks of information and identify physical persons even when each element comes from anonymous sources (profiling). Therefore, the need for a framework to address the impact of OSINT on individual rights is quite evident in the literature (Rahman and Ivens 2020). So much so that several European projects dealing with the issue of privacy in OSINT investigations have been funded over the years.^3^ These projects aimed at developing platform solutions for the retrieval, analysis, management and dissemination of OSINT across Law Enforcement Agencies (LEAs) in different policing contexts, keeping track of the evolving European data protection framework (Cuijpers 2013; Casanovas et al. 2014).

As a result, much literature has been produced in the practitioner (Dupont et al. 2011; Amardeilh et al. 2013; Ortiz-Arroyo 2015) and GELSI strands. Although the latter focuses mainly on the specific OSINT software tools developed as part of the European research projects, the main ethical and legal concerns are also laid out. The crucial issue is the impact of OSINT investigations on individuals’ privacy, and the go-to framework to address these concerns is Privacy by Design (PbD). A concept mainstreamed by Cavoukian and Borking in the 1990s (Hustinx 2010, 253) and later adopted in data protection legislation (see, for instance, Art. 25, GDPR 2016), PbD is linked to the creation of Privacy Enhancing Technologies (PETs) that are aimed at safeguarding individual privacy during the design of data analysis software and platforms (Cuijpers 2013). Several principles have been suggested to achieve this behavior by default. Colesky et al. (2016) summarize them in eight tactics that should guide PbD strategies: minimize, hide, abstract, separate, inform, control, enforce, and demonstrate. These tactics cover both the data collection and use phases, and include essential tools, such as encryption, anonymization, data aggregation, informed consent, and auditing strategies.

Additionally, Koops et al. (2013) propose two ways of directly incorporating PbD principles into OSINT platforms: revocable privacy and enterprise privacy policies. The first one aims at enforcing the data minimisation strategy by allowing access to personal data “only if a predefined condition has been met” (Koops et al. 2013, 681). This could be achieved either through spread responsibility, i.e., relying on a third party to verify whether the condition(s) has occurred and release the relevant data, or through a self-enforcing architecture, i.e., a set of hard-coded rules that would grant access to the relevant data automatically, if triggered by some precondition.

Enterprise privacy policies use technology itself to implement privacy rules. Specifically, they require a policy markup language to define the required data management and access rules (Koops et al. 2013, 683). This way, more sophisticated legal compliance mechanisms can be embedded within machine code.

Another critical aspect, when considering the legal implications of OSINT platforms, is creating and maintaining specialized ontologies designed to automate parts of the analysis, such as document summarisation or entity extraction. These ontologies need to be specified correctly to be interoperable between agencies and need frequent updating to keep up with evolving regulatory frameworks. Ontologies are formally defined as “(meta)data schemas, providing a controlled vocabulary of concepts, each with an explicitly defined and machine processable semantics” (Maedche and Staab 2001). They play a crucial role in designing Semantic Web Regulatory Models (SWRM), which encode norms, rules and ethical principles into machine-readable regulation, which can then be applied across different organizations and countries (Casanovas 2015). However, ontology regulation is only partly addressed when it comes to OSINT. As Casanovas et al. (2014) put it, “[t]here are no neutral ontologies. They have a purpose and a particular shape, and need to be regularly updated”. Therefore, a practical framework for the definition (and maintenance) of ontologies for OSINT analysis is needed in the GELSI literature, especially considering the varied nature of OSINT sources and the potential deceitful use the opponent may make of it.

Other authors shift the focus from PETs to other legal devices to address the OSINT “privacy paradox” of information being freely available (not private) but also sometimes extremely sensitive and personal (therefore private). One hypothetical solution provided in the literature draws from Nissenbaum (2004). It considers privacy as contextual integrity, namely the idea that no area of life is exempt from privacy expectations and that every situation has contexts regulated by explicit or even implicit norms that, if violated, result in a breach of privacy. This idea, coupled with the extension of the concept of home to the digital realm, where each user can decide who has access to their “personal cyberspace”, would ensure that privacy is at least partly safeguarded against malicious use of personal data (Ten Hulsen 2020).

Indeed, at the moment, the availability of OSINT to inexperienced (or malicious) activists can lead to unethical and possibly even illegal behavior, such as the sharing of private information online or the misidentification of individuals involved in illegal activity (Belghith et al. 2022). This should be a source of concern for organizations and activist networks, as the infringement of the ethical code surrounding the OSINT community could have severe consequences regarding public safety and national security. Moreover, as Shere (2020a) recently argued in a survey of OSINT analysts, the General Data Protection Regulation (GDPR) has failed to provide a significant change in OSINT gathering capabilities, with any meaningful change only being due to the updating of privacy settings by social media companies. These and other elements of concern for individuals and groups involved in OSINT investigations have been summarized by Hu (2016) in five major ethical concerns of OSINT practice. They include the origin and intent of sources, which should be carefully vetted, as they could bias the resulting analysis (more on this misinformation aspect in the following sections); the distribution of unclassified yet still sensitive information, which could harm the people involved; and the over-reliance on automated analysis, which could lead to mistakes if left unchecked. Moreover, the mosaic effect, whereby data subjects can be identified by integrating different data sources and the excessive publicity generated by successful OSINT investigations are also sources of concern for the analyst.

These aspects also find their way into the legal realm. Indeed, there is a difference between OSINT as *intelligence* and OSINT used as *evidence* in a criminal proceeding (Sampson 2016). While almost any information can be considered intelligence when it serves a specific purpose, in the latter case, the evidential material must answer further questions of admissibility and weight. Specifically, the evidence must be proven relevant to a fact in question, and its reliability must be established before it can be accepted. Looking at the general case of the signatories of the European Convention on Human Rights (ECHR), Sampson (2017) identifies some procedural issues in using OSINT as evidence, mainly concerning the fairness principles embodied in the ECHR. In general, aside from country-specific legislation, disclosure of evidence to defendants is expected in criminal trials. Moreover, the hearsay nature of many OSINT materials could lead to their rejection in court. According to the author, three main factors determine the admissibility of OSINT data as evidence in criminal trials. First, the provenance of the material requires that the data source be clearly identified, and that the collection procedure be lawful. In the specific case of digital OSINT, Lyle (2016) provides some examples of unlawful OSINT collection by law enforcement, such as impersonating someone on social media, which require specific legal authority and would compromise the admissibility of OSINT material in court. Second, data integrity concerns the reliability of the evidence itself. If the data could have been easily tampered with [see, for instance, the case of deep fakes in Koenig (2019)], its admissibility and weight would be in question. The last aspect to be considered is the reliability of the author providing the evidence. If the material comes from an anonymous source or the author cannot corroborate the evidence, the data will likely be rejected during the trial (Sampson 2017).

While most of the scholarship at the micro-level is concerned with data subjects and how to safeguard their rights appropriately, there is yet another important line of research dealing with the potential harms occurring to OSINT practitioners conducting analyses. One of the main risks is known as vicarious trauma, namely the psychological trauma caused by handling materials portraying violence. While safety procedures and recommendations to reduce the impact of vicarious trauma, such as removing sound and reducing video resolution, have long been around in the OSINT community (Parry 2017), automated frameworks to identify and tag potentially sensitive digital content have also been proposed (Breton et al. 2021).

## The GELSI of OSINT at the macro-level

At the macro-level of analysis, the focus shifts from individuals, software solutions, and organizations to broader debates about the social and political implications of OSINT. Here, we find papers dealing with OSINT in a more abstract sense, not limited to the intelligence studies domain, but encompassing the broader social science literature. This literature partly emerged as a reflection on the experience of the open-source hacking communities. Born and nurtured around a shared ethos of “transparency, truth and trust” (Steele 2012), such communities flourished in the early years of the Internet. They were responsible for developing several open-source projects, from operating systems to web servers. Through the trusted user system, a large community of programmers was able to update code and share information almost in real-time while working on a well-defined project, thus cementing the idea of the Internet as a “new ecology of interconnected ideas” (Glassman 2013, 682).

As its software analog, Open Source Intelligence has been presented as a way of democratizing access to information and fostering citizen activism, removing any intermediary and allowing a collaborative search for the truth. Thanks to a shared moral code among OSINT practitioners, which “prioritizes transparency and accountability, frowns upon the use of subterfuge, and limits investigations to passive reconnaissance” (Belghith et al. 2022, 2), some in the techno-libertarian fringe argue that OSINT will be able to increase oversight over secret government activity and therefore reduce the invasive reach of security agencies, eventually leading to an “Open Source Everything” society (Steele 2012).

Interestingly, this idea is tied to a long-standing debate about the nature and evolution of human cognition. Glassman and Kang (2012) interpret OSINT as a bridge between the two intelligence categories defined by Horn and Cattell (1967): fluid intelligence and crystallized intelligence. While the former represents a more intuitive approach to problem-solving based on abstraction and pattern detection, as seen in childhood, the latter defines intelligence as the ability to solve problems by applying methods and tools already learned through experience and is, therefore, more prominent in the later stages of human development. Thus, crystallized intelligence is typically applied to known problems and uses problem-solving strategies that are well-known and culturally shared, while fluid intelligence is relied upon when facing new problems requiring abstract thinking and mental flexibility. According to the authors, OSINT can bring insights and creativity from fluid intelligence into the realm of a more codified and community-based cultural intelligence. This process is enabled by the free access to the web and the horizontal nature of open-source information, allowing for novel approaches to investigative work that can be crowd-sourced across the (virtual) community. As the authors put it:

*OSINT is controlled exploration that is open to new and different connections and possibilities combined with focused problem solving. OSINT promotes goal directed activity that is capable of transcending social and cultural boundaries* (Glassman and Kang 2012, 677, emphasis in text)

The ability to overcome cultural boundaries is ensured by the continuous flow of unfiltered information available to the citizen/analyst and constitutes a step forward in creating a *Smart Nation*, which, in the words of Robert David Steele:

educates and enables every citizen to be a collector, producer, and consumer of legal, ethical, open-source intelligence, and also to be a vibrant member of the authentic intelligence community of the whole—humanity connected as one, thinking as one, acting as one (Steele 2012).

A collection of *smart nations* would then build toward a global noosphere, a worldwide community based on “multinational information sharing and sense-making” (Steele 2012). Such a radical societal transformation finds significant parallels in the smart governance literature, where meaningful social change is reached through a dynamic dialog between state and non-state actors (Willke 2007). In this scenario, OSINT is seen as a critical tool in leveling the playing field and ensuring a degree of transparency conducive to this dialog.

While the above articles highlight how the nature of OSINT makes it a crucial tool for democratic oversight, this very nature can also be seen as a threat to citizens’ rights when authorities exploit it to increase social control (Wells 2016). Concerns about increased state surveillance and profiling have long been expressed in the literature (Eijkman and Weggemans 2012), together with the opacity in the analysis and OSINT-based decision-making by state authorities and private companies. Indeed, it has also been shown that the growing public awareness of state surveillance practices and fear of profiling can lead users to contemplate withholding or even falsifying personal information shared online (Bayerl and Akhgar 2015). Similarly, awareness of OSINT tools and capabilities has been linked to more robust security behavior in IT professionals (Daniels 2016). Consequently, efforts to educate users about the ramifications of their online activities have emerged, and are likely to reinforce this trend (Parry 2017; Young et al. 2018).

This, coupled with the blossoming of disinformation strategies in domestic and international affairs, contributes to the muddying of the OSINT waters and has obvious implications for the reliability of OSINT data collected during investigations (Miller 2018; Olaru and Ştefan 2018). “Open” does not equal “true”, also when it comes to OSINT, and in the absence of a shared standard to detect and filter out falsified information, each analyst has so far relied on their own sectorial experience to validate the intelligence collected. As McKeown et al. (2014) point out, this can lead to significant differences in reporting accuracy across analysts, as some may deem a source reliable while others may not. This could lead to a vicious cycle of distrust where OSINT sources get increasingly polluted with unreliable information that, if not promptly identified, could sway the decision-making process.

## Future research directions: the GELSI of AI and OSINT

Listed at the bottom of Fig. 6 are a few suggestions for future research on the interplay between OSINT and AI at the micro and macro levels. These are developments we find most likely to occur in the upcoming OSINT literature, as AI algorithms become closely intertwined with everyday OSINT practice.

At the *micro-level*, the OSINT-GELSI scholarship has been concerned so far with the knowledge retrieval and management aspects of the OSINT pipeline. However, current legislation gives little importance to the phases of *analysis* and *use* of machine-gathered OSINT. Building on the work by Broeders et al. (2017) and others in the AI auditing literature, increased effort should be devoted to developing a theoretical framework to regulate those aspects in the data analysis phase which could impact algorithmic performance (i.e., variable selection, model weights, optimisation algorithms, etc.).

Moreover, more comprehensive scholarship on *algorithmic opacity* could inform future literature on OSINT and AI. Burrell (2016) identifies three main sources contributing to the overall lack of transparency in how AI algorithms are employed. The first one, corporate secrecy, relates to the intentional concealment of the inner workings of algorithms by companies to safeguard their products. Proposed solutions are mainly on the legislative side, and involve developing disclosure and auditing frameworks where trusted third parties would be tasked with reviewing code and ensuring that appropriate ethical standards are met (Pasquale 2015; Lu 2020). The second source of opacity, technical literacy, refers to the specialized nature of writing and reading computer code, which makes it difficult for end-users and regulators to understand fully the mechanics and results of AI algorithms. Increasing computer literacy and “computational thinking” (Lee et al. 2011) among critical sectors of civil society is seen as an essential step in countering this source of opacity. Finally, the black box structure of many machine learning models makes it difficult to interpret results correctly, even for practitioners. The reliance on multi-component systems only increases the complexity of the overall infrastructure, further increasing the time and effort required for auditing. Different solutions have been proposed to deal with this complexity. They are mainly focused on technical tools that can reduce the dimensionality of the data, create metrics that can evaluate the fairness of algorithms and provide graphical visualizations of relationships between key variables to aid the analysis (Dwork et al. 2012; Paudyal and William Wong 2018).

Transparency is even more crucial when it comes to OSINT data. Indeed, while many auditing strategies apply to all data types, open source data must satisfy more stringent validity requirements and should be specifically targeted as a priority when devising regulatory strategies. Moreover, since veracity assessment via machine learning algorithms has been growing in recent years (Manzoor and Singla 2019), it is also likely that the applied literature on misinformation detection will play an important role in designing strategies to filter out irrelevant data, thus preventing or at least reducing biased outcomes during analysis. There have already been some attempts at designing frameworks for automatic veracity assessments of open source information (see, for instance, Lozano et al. 2015). However, using AI to validate data also raises important ethical questions which have been largely left unanswered, as Lozano et al. (2020) point out. Some of these questions relate to the allocation of responsibilities when data is mislabelled, together with the optimal procedures to determine veracity in the first place.

Considering the original Open Source movement ethos, one could envisage oversight mechanisms that are themselves open source. For instance, publishing the source code of the algorithms employed during online investigations, together with the updated datasets and their respective veracity assessments. However, this would be not just extremely unlikely, given the secretive nature of most OSINT investigations. It could also become counterproductive, as data published by state authorities could be exploited as intelligence by actors interested in attacking the enemy’s infrastructure.

As for broader privacy concerns, AI algorithms could threaten the PbD tactics outlined in Sect. 5 and other solutions, such as contextual integrity approaches, specifically to the data retention and minimisation principles. As it turns out, analysts may be tempted to keep users’ information stored in their databases, especially since data that appear useless today may prove relevant in the future. This phenomenon, known as function creep in the literature (Koops 2021) has already been observed in OSINT investigations (Trottier 2015), and is likely to become more prominent as the growing computing power allows AI algorithms to process an enormous number of features.

Integrating AI tools into data acquisition and analysis routines also provides legal challenges when moving from intelligence collection to evidence presentation. It has already been observed how cognitive and technical biases influence digital OSINT collection by narrowing the search space based on the analyst query or the search engine’s ranking of results (McDermott et al. 2021, 92–93). Thus, relying on automated collection and analysis could amplify these biases and raise questions of admissibility if intelligence is used as evidence in criminal trials.

At the macro-level, research about the GELSI of OSINT should address how AI-powered OSINT may invalidate the oversight potential of publicly available information (as claimed by the Open Source movement). Indeed, the availability of AI algorithms increases data processing capabilities (and, to a lesser extent, disinformation detection), and it does so asymmetrically. Standard OSINT gathering and analysis methods focus on selecting appropriate data sources and require only a limited grasp of technological solutions. However, automating collection and analysis involves a much deeper understanding of AI algorithms. This knowledge is unequally distributed among potential OSINT users and favors those with access to larger computing power and better expertise. Most commercial solutions available to the public (whether free or subscription-based) heavily use web crawlers that automate intelligence collection (Pastor-Galindo et al. 2020). However, currently available software does not provide AI algorithms in the analysis phase, which must be coded separately. Thus, in the future, actors with access to AI solutions for analyzing open source data will hold a comparative technological advantage over the rest, being able to process and classify a much larger quantity of data in considerably less time. This conflict could manifest between different state and non-state actors simultaneously, threatening the oversight role of OSINT data and exacerbating its surveillance and social control aspects. Moreover, the prevailing trend toward intelligence outsourcing to private companies could create even more friction between the public and private sectors, as already noted in Bean (2011), even more so when better algorithms yield better (intelligence) products.

Yet another essential issue that needs to be addressed in the OSINT-GELSI literature is whether AI will prevent OSINT from maintaining the equilibrium between fluid and crystallized intelligence. While it is true that OSINT can push analysts beyond traditional investigative routes that are determined by crystallized knowledge through its fluid intelligence properties, it is also true that current AI systems follow problem-solving strategies that are closely related to crystallized intelligence. Supervised machine learning is built to allow the computer algorithm to learn variable dependencies from a sanctioned body of knowledge (the *labeled data*) to identify similar patterns in unseen data. Indeed, despite many attempts at developing new learning models for abstract reasoning, it has been argued that AI algorithms have only been able to achieve crystallized intelligence because they are designed to tackle only a given task (or a limited set of tasks), without being able to generalize their knowledge to previously unseen problems (Davidson and Walker 2019; van der Maas et al. 2021, 5). Consider, for example, the case of digital media. While it would be possible to train an algorithm to detect enemy combatants from a digital open source, it is unlikely that the same algorithm will be able to infer other significant intelligence, such as geolocation data, if it has not been trained to recognize those data. At the same time, without specialized training, it would prove almost impossible to assess the context of the media data and determine, for instance, whether it was an excerpt from a movie or footage from an unrelated military exercise disseminated for disinformation purposes. Therefore, any OSINT analysis aided by AI algorithms heavily relies on a crystallized intelligence approach, and risks losing some of the abstract intuition of its fluid component every time a task requires “higher levels of behavioral flexibility and adaptivity” (Schilling et al. 2019). Furthermore, over-reliance on OSINT software like that mentioned in Sect. 5 will likely worsen this condition. Indeed, when OSINT data are automatically crawled and analyzed, only significant matches are returned through the analyst queries, and some crucial detail may go missing altogether. This problem has already emerged in the literature and will remain dominant (Odom 2008, 325). Eldridge et al. (2018) argue that OSINT analysis should never get rid of its human component, and that “joint cognitive systems” (Eldridge et al. 2018, 22) should be designed to strike the optimal balance between analysts and algorithms.

## Conclusions

This article provides a systematic review of the GELSI literature on OSINT. The OSINT-GELSI scholarship can be broadly divided into two main categories, namely the micro and macro levels of analysis. At the micro-level, authors look at the impact of OSINT on individuals and organizations, tackling privacy issues and oversight mechanisms within the development of software for the exploitation of OSINT resources. At the macro-level, the main focus is on the social and political implications of the production and availability of OSINT data. Some articles analyze how increased awareness of OSINT tools and capabilities modifies online habits, with many users deciding to share less personal information or turn to anonymity to limit their online exposure. Meanwhile, other articles tie into the broader open-source movement literature, reflecting on the role of OSINT in the relationship between fluid and crystallized intelligence, leading to the growing democratization of intelligence and the creation of more transparent societies.

Research dealing with OSINT augmented by AI algorithms is emerging and will likely become predominant in future OSINT-GELSI scholarship. At the micro-level, greater emphasis should be placed on the AI-auditing literature. In particular, more research is needed to deal with regulatory strategies to oversee and direct the processes of information gathering and analysis carried out through data mining and machine learning techniques. This is increasingly pressing as more data are collected, processed, and labeled automatically for further use. Due to its many reliability issues, particular attention should be given to OSINT data. One key feature that should be targeted is using OSINT for deliberate disinformation, which could easily sway even the more sophisticated algorithms and pollute datasets used to train them. Efficient diagnostic techniques should be designed to tackle this issue and minimize errors, thus reducing the risk of incorporating bias into OSINT investigation results (Lozano et al. 2020).

At the macro-level, the OSINT-GELSI literature should look at the role of AI in shaping the relationship between OSINT and society. For instance, while it is believed that OSINT constitutes a bridge between crystallized and fluid intelligence, the very nature of machine learning algorithms places AI in the former group. This means that the increasing integration of AI in OSINT tools relying on automated rather than human-centered analysis could shift the balance toward a crystallized approach to OSINT problems. Another critical ramification to be considered at the macro-level is the changing nature of the *owners* of OSINT tools, which become exclusive providers of intelligence for public and private use.

As a general recommendation, the OSINT-GELSI scholarship should be more aware of the results of the OSINT practitioner literature to get a grasp of the emerging trends and be able to react promptly to potential ethical challenges raised by them. As we have argued in the previous sections, OSINT is not only improved by technology. Its scope is expanded as new sources of information become public. However, the more complex the data (and the larger the data pool), the more complex collection and analysis will become. As a result, state and non-state actors with access to enough computing power and the right expertise will have a comparative technological advantage over the others. Thus, a comprehensive GELSI analysis of OSINT tools and techniques cannot ignore the latest trends in the applied literature and should try to anticipate them by looking at other fields where the same issues have already become manifest. Increasing integration between the GELSI and applied domains is not just desirable but necessary to address current and future ethical issues.

## Data Availability

The data that support the findings of this paper is available upon request.

## Footnotes

1 Intelligence cycles are modeled in different ways by different intelligence organization, in this article we follow the model proposed in the US Joint Intelligence report (Defense Technical Information Center (DTIC)—Department of Defense 2013).

2 The acronym GELSI expands another acronym, ELSI (Ethical, Legal and Social Implications), which originated in the fields of biotechnology and genetics. This research field “addresses the relationship between the new and emerging techno-sciences and society” (Zwart and Nelis 2009) and involves predicting and possibly proposing solutions to new challenges posed by their interaction.

3 Projects like the *Versatile InfoRmation Toolkit for end-Users oriented Open Sources explOitation* (VIRTUOSO, 2010–2013), the *Collaborative information, Acquisition, Processing, Exploitation and Reporting for the prevention of organized crime* (CAPER, 2011–2014) and the *Maritime Integrated Surveillance Awareness* (MARISA, 2017–2020) are some of the most prominent examples.
